# Specificity protein 1-mediated ACSL4 transcription promoted the osteoarthritis progression through suppressing the ferroptosis of chondrocytes

**DOI:** 10.1186/s13018-023-03673-0

**Published:** 2023-03-10

**Authors:** Wen He, Xuchao Lin, Kangyao Chen

**Affiliations:** Department of Orthopaedics, Fuzhou Second Hospital, No. 47, Shangteng Road, Cangshan District, Fuzhou City, 350007 Fujian Province China

**Keywords:** Osteoarthritis, Ferroptosis, Acsl4, Sp1

## Abstract

**Background:**

Chondrocytes are the main cell damage type involved in the occurrence and development of osteoarthritis (OA). Ferroptosis has been confirmed to be related to many degenerative diseases. This research aimed to explore the role of Sp1 and ACSL4 in ferroptosis in the IL-1β-treated human chondrocyte cells line (HCCs).

**Methods:**

The cell viability was detected with CCK8 assay. The ROS, MDA, GSH, and Fe^2+^ levels were assessed with corresponding detecting kits. The Col2a1, Acan, Mmp13, Gpx4 and Tfr1 levels were determined by RT-qPCR assay. Western blot was conducted to evaluate the Acsl4 and Sp1 levels. PI staining was carried out to analyze the cell death. The double luciferase report was conducted to verify the interaction between Acsl4 and Sp1.

**Results:**

The results showed that IL-1β stimulation elevated the LDH release, cell viability, ROS, MDA and Fe^2+^ levels and declined the GSH levels in the HCCs. Additionally, the mRNA levels of Col2a1, Acan, and Gpx4 were prominently decreased, while Mmp13 and Tfr1 were prominently elevated in the IL-1β stimulated HCCs. Furthermore, Acsl4 protein levels were upregulated in the IL-1β-stimulated HCCs. Both Acsl4 knockdown and ferrostatin-1 treatment neutralized the role of IL-1β in the HCCs. What’s more, Acsl4 was transcriptionally regulated by Specificity protein 1 (Sp1). Sp1 overexpression enhanced the Acsl4 levels and Sp1 knockdown declined it.

**Conclusion:**

Upregulation of Sp1 activates Ascl4 transcription and thus mediates the occurrence of ferroptosis. Hence, Acsl4 may be a therapeutic target for intervention of OA.

## Introduction

Osteoarthritis (OA) is a chronic disease characterized by articular cartilage degeneration, cartilage ossification and secondary hyperosteogeny [1, 2]. OA most often affects the knee joint, resulting in severe joint deformity and joint dysfunction. In severe cases, it can cause disability, which directly affects the quality of life of the middle-aged and elderly [3]. The pathogenesis of OA is complex, and inflammatory factors, metabolism and other factors are closely related to OA. As the only cellular component of cartilage, chondrocytes are the main cell damage type involved in the occurrence and development of OA [4]. Research showed that chondrocyte injury can be divided into cell necrosis, apoptosis and autophagy cell death [5].

In 2012, S. J. Dixon et al. [6] first reported a new form of cell regulated death, which is different from other forms of cell death in morphology, biochemistry and gene, and named it ferroptosis. Ferroptosis is an iron-dependent non-apoptotic cell death characterized by inactivation of antioxidant enzyme glutathione peroxidase 4 (GPX4) and accumulation of lipid reactive oxygen species [7]. So far, ferroptosis has been confirmed to be related to many degenerative diseases (such as Alzheimer's disease, Parkinson's disease, renal degeneration), carcinogenesis, intracerebral hemorrhage, traumatic brain injury, ischemia–reperfusion injury and stroke [8–11].

Acyl coenzyme A synthetase long-chain member 4 (ACSL4), a member of the long-chain acyl CoA synthetase family, catalyzes the synthesis of acyl CoA in vivo as the first step of fatty acid catabolism [12]. Previous study found that ACSL4 is a key gene in the ferroptosis pathway, which can synthesize arachidonic acid and adrenal acid into arachidonic acid CoA and adrenal acid CoA, respectively, to participate in membrane phospholipid synthesis [13]. Under the treatment of RSL3, a ferroptosis inducer, the long-chain polyunsaturated fatty acids on the membrane are easy to be oxidized, leading to ferroptosis [14]. However, there are few studies on ACSL4-mediated ferroptosis in the progress of OA.

Specificity protein (Sp1) is a member of the SP/KLF transcription factor family, which is located in the nucleus [15]. Sp1 transcription factor is widely expressed in vivo and is involved in regulating the expression of many genes in mammalian cells [16]. Sp1 can interact with a series of proteins, including other transcription factors and epigenetic regulatory factors [17, 18]. Chromosome mapping studies have shown that there are at least 12,000 Sp1 binding sites in the human genome, which are related to genes involved in most cell processes [19]. A recent study has demonstrated the Sp transcription factor family was closely related to the OA progression, whose function in osteosarcoma development was confirmed by bioinformatic analysis [20].

Therefore, this research aimed explore the functions of Sp1 induced ferroptosis in OA progression. We hypothesized that Sp1 was upregulated in OA, which activates the transcription of Ascl4 to induce ferroptosis of.

## Materials and methods

### Cell culture and treatment

Human chondrocyte cells line CHON-001 (HCCs) were purchased from ATCC. Cells were maintained in DMEM medium (Gibco, GI, USA) containing 10% fetal bovine serum (FBS, Gibco) and 1% penicillin–streptomycin. Then the cells were incubated at 37 °C with an atmosphere of 5% CO_2_ and 95% humidity. Next, the HCCs were treated with IL-1β(10 ng/ml) for 24 h to establish an OA model in vitro. Additionally, in order to inhibited ferroptosis, 1 µM of Ferrostatin-1 (Fer-1) was used to treat the HCCs.

### Cell transfection

Small interfering RNA Acsl4 (si-Acsl4 1#, si-Acsl4 2#), small interfering RNA Sp1 (si-Sp1 1#, si-Sp1 2#) and their controls (si-nc), Sp1 overexpression vector (oe-Sp1) and empty vector (oe-nc) were all obtained from GenePharma (Shanghai, China). HCCs were seeded in 6-well plates (2 × 10^5^ cells/ well). The plasmids were transfected into HCCs using Lipofectamine 3000 reagent (Life Technologies, USA), with all procedures following the manufacturer’s protocol. The siRNA primer sequence:si-Acsl4 1#, 5′- GGGAGUGAUGAUGCAUCAUAGCAAU-3′;si-Acsl4 2#, 5′- GGCUUCCUAUCUGAUUACCAGUGUU-3′;si-Sp1 1#, 5′-AUGAUCUGUAUUUGACCAGTT-3′;si-Sp1 1#, 5′-UAUUUGGGAUGAUCUGUUGGUTT-3′;si-nc, 5′-ACGUGACACGUUCGGAGAATT-3′.

### Cell viability determination

A CCK-8 kit (Beyotime, Shanghai, China) was purchased to access cell viability of HCCs. In short, cells in each group were seeded in 96-well plates (2 × 103 cells/ well) and cultured for 48 h. Then, HHCs were treated with CCK8 solution reagent for 2 h, and 450 nm absorbancy was chosen in a microreader to assess cell viability.

### LDH release, MDA, ROS, GSH, and Fe^2+^ levels determination

The LDH release, MDA, ROS, GSH, and Fe^2+^ levels of the HCCs in each group were assessed the with Corresponding kits purchased from Nanjing Jiangcheng Bioengineering Institute (Nanjing, China). All procedures were followed the manufacturer’s protocol.

### PI staining assay

HCCs were washed with PBS and fixed with cold 70% ethanol at 4 ℃ for 60 min. Then the cells were stained with 1 mL PI (sigma) at 4 ℃ for 30 min. The samples were counterstained with DAPI. The fluorescence signal was observed by a fluorescence microscope.

### Immunofluorescence staining

The cells were fixed with 4% paraformaldehyde for 15 min and washed with PBS. Next, the cells were permeated with 0. 2% Triton X-100 for 5 min, and cultivated with 5% BSA for 30 min. After the blocking solution was discarded, the cells were incubated with anti-Ascl4 overnight. The next day, after rewarmed for 30 min, the cells were incubated with secondary antibody for 1 h. The nucleus was stained with DAPI for 4 min in darkness. Images were acquired on a fluorescence Nikon (DIAPHOT) fluorescence microscope.

### qRT-PCR

qRT-PCR was conducted to measure the expression levels of Col2a1, Acan, Mmp13, Gpx4, and Tfr1. Total RNA of cells was extracted by Trizol (Beyotime), and cDNA was synthesized using the total RNA and a reverse transcription kit (Vazyme, Nanjing, China). Afterward, the cDNA was used as the template for qRT-PCR amplification (Bio-Rad, CFX-96) with a SYBR Green Mix kit (Vazyme). Relative gene expression was calculated using the 2^−ΔΔCt^ method, and GAPDH was selected as the internal control. PCR primer sequences were listed below (5′ → 3′): Col2a1, F:TGGACGATCAGGCGAAACC and R: GCTGCGGATGCTCTCAATCT; Acan, F: ACTCTGGGTTTTCGTGACTCT and R: ACACTCAGCGAGTTGTCATGG; Mmp13, R: TCCTGATGTGGGTGAATACAATG and R: GCCATCGTGAAGTCTGGTAAAAT; Gpx4, F: AGTACAGGGGTTTCGTGTGC and R: CATGCAGATCGACTAGCTGAG; Tfr1, F: ATGTTGGATGGGTAGCCAAAG and R: TTCGAGAGCGCAAATCTTCTG; Acsl4, F: CATCCCTGGAGCAGATACTCT and R: TCACTTAGGATTTCCCTGGTCC; GAPDH, F: TGTGGGCATCAATGGATTTGG and R: ACACCATGTATTCCGGGTCAAT.

### Western blot analysis

Total protein was extracted with RIPA buffer (Beyotime) and quantified by the BCA protein detection kit (Beyotime) in accordance with the protocols. Proteins were electrophoresed on 10% SDS-PAGEs and transferred to PVDF membranes (Millipore). Afterward, membranes were blocked with 5% nonfat milk for 1 h and treated with the specific primary antibodies against Acsl4 (1:1000, abcam), Sp1 (1:200, abcam) and GAPDH (1:2000, abcam) at 4 ℃ overnight. Following conjugation to HRP-labeled secondary antibody, the bands were visualized with a super ECL kit following the instructions (Beyotime).

### Co-Immunocoprecipitation (CO-IP)assay

The combination between Ascl4 and Sp1 in HCC cells was detected according to the instructions of CO-IP kit (Biomars Technology Development Co., Ltd, Beijing, China). IgG was used as the control, 5 μg Ascl4/Sp1 antibody was added into the reaction tube. Then the protein levels of Ascl4/Sp1 and GAPDH in anti-Ascl4/Sp1 and IgG precipitation were detected by Western blot. IgG group is used as control.

### Luciferase reporter assay

HCCs were cultivated in 24-well plates and cotransfected with the Sp1 overexpressing plasmids and the report vector carrying wild type or mutant promoter of Ascl4. 48 h after transfection, Luciferase activities were measured using a Dual-Luciferase Reporter Assay System (Promega).

### Statistical analysis

The experimental data were analyzed by GraphPad Prism and was represented by mean ± SD. The comparison between two groups was compared with *T* test. One-way ANOVA followed by Tukey’s post hoc test was used to compare the difference between multiple groups l. *P* < 0.05 means the difference is statistically significant.

## Results

### Ferroptosis exists in IL-1β-treated cells

**Fig. 1:**
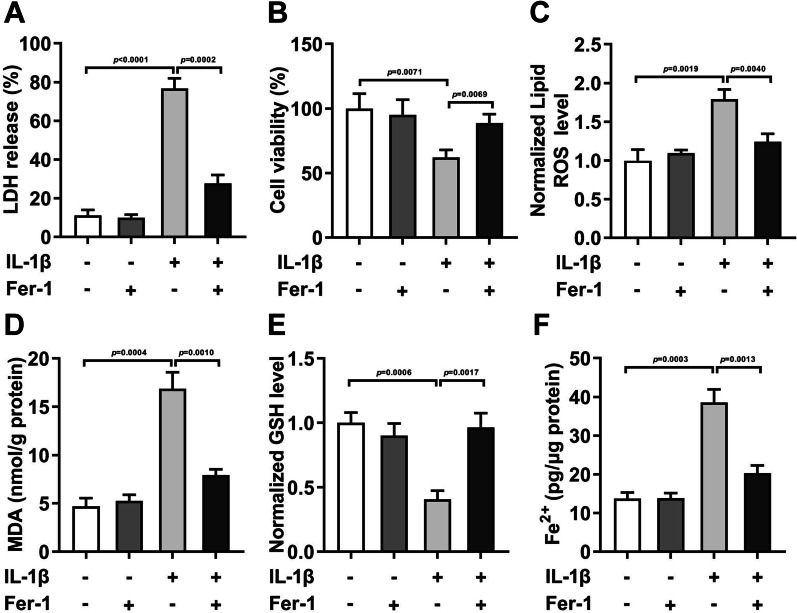
Ferroptosis occurred in IL-1β-treated cells. After IL-1β and Fer-1 treatment in the HCCs, the LDH release **A** was measured with LDH Toxicology Kit. Cell viability **B** was detected by CCK-8 assay. The ROS (**C**), MDA (**D**), GSH (**E**), and Fe^2+^ **F** levels were assessed with Corresponding kits

In the HCCs, after IL-1β treatment, the LDH release (Fig. 1A, *P* < 0.0001), ROS (Fig. 1C, *P* = 0.0019), MDA (Fig. 1D, *P* < 0.0020), and Fe^2+^ levels (Fig. 1F, *P* < 0.0001) were dramatically enhanced, while cell viability (Fig. 1B, *P* = 0.0058) and GSH levels (Fig. 1E, *P* = 0.0006) were dramatically declined. After Fer-1 treatment, the role of IL-1β in the HCCs were neutralized. Additionally, Fer-1 showed no effects on the normal HCCs.

### IL-1β treatment induced the changes in ferroptosis-related genes

**Fig. 2:**
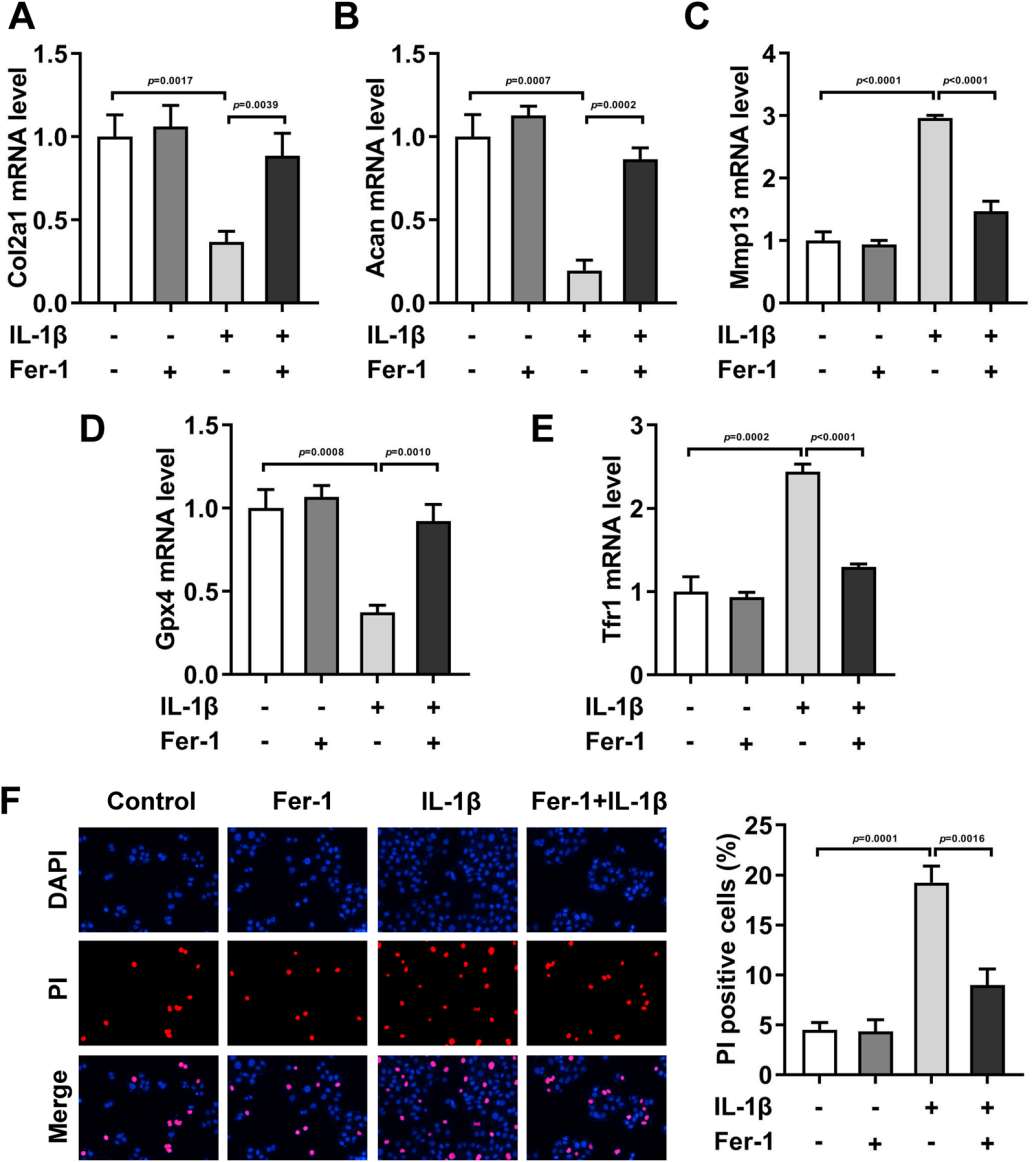
IL-1β treatment induced the changes in ferroptosis-related genes. After IL-1β and Fer-1 treatment in the HCCs, the mRNA levels of Col2a1 (**A**), Acan (**B**), Mmp13 (**C**), Gpx4 (**D**) and Tfr1 **E** were determined by RT-qPCR assay. **F** The cell death was analyzed by PI staining

Then, in the HCCs, after IL-1β treatment, the mRNA levels of Col2a1 (Fig. 2A,* P* = 0.0017), Acan (Fig. 2B, *P* = 0.0007), and Gpx4 (Fig. 2D, *P* = 0.0008) were prominently depleted, while mRNA levels of Mmp13 (Fig. 2C, *P* < 0.0001), Tfr1 (Fig. 2E, *P* = 0.0002), and PI-positive cells (Fig. 2F, *P* = 0.0001) were prominently elevated. After Fer-1 treatment, the role of IL-1β in the HCCs was neutralized. Additionally, Fer-1 showed no effects on the normal HCCs.

### Acsl4 was overexpressed in IL-1β-treated HCCs

**Fig. 3:**
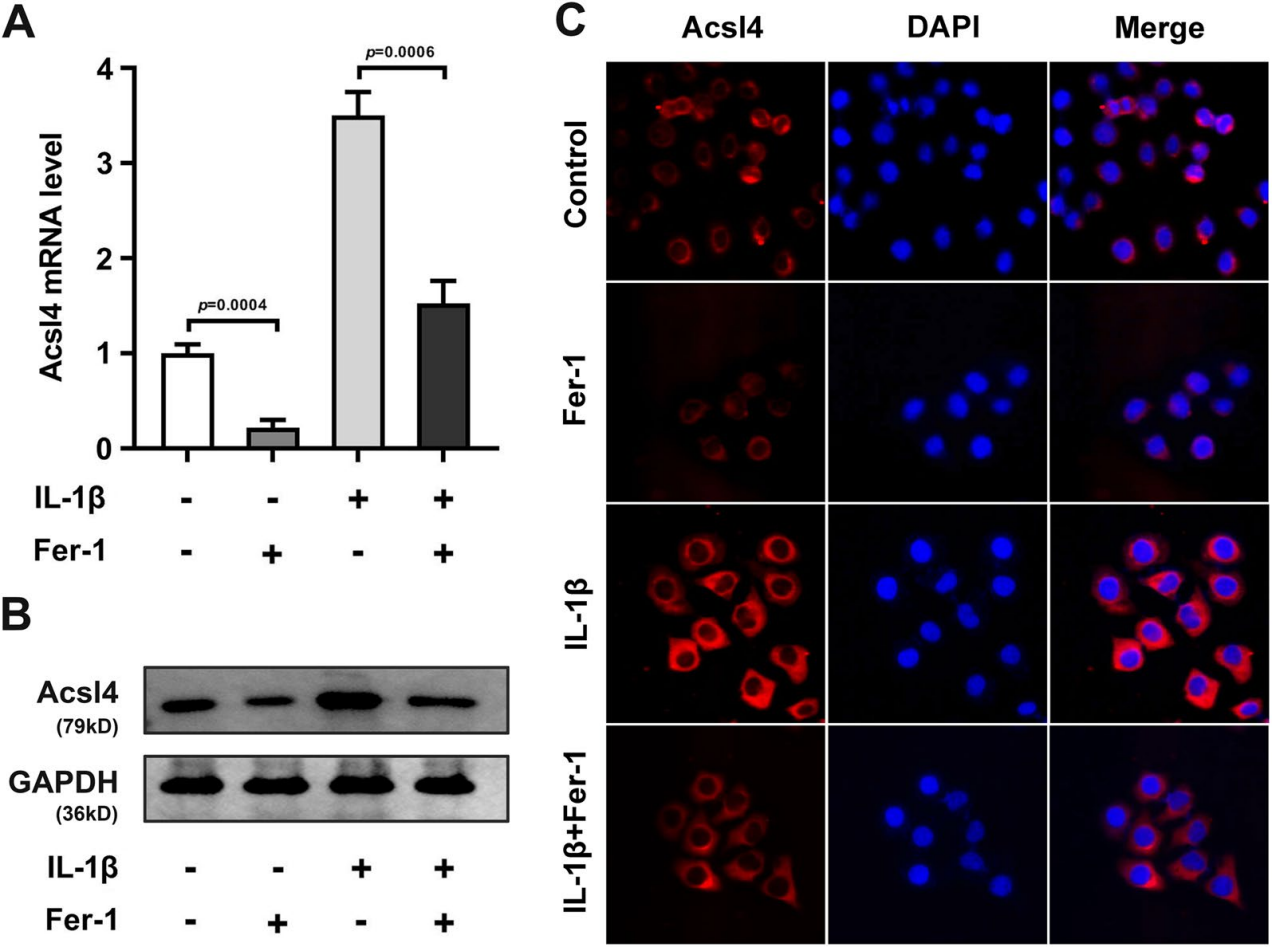
Acsl4 was overexpressed in IL-1β-treated HCCs. After IL-1β and Fer-1 treatment in the HCCs, the Acsl4 levels were analyzed by PCR (**A**), Western blot (**B**), and immunofluorescence (**C**)

Acsl4 has been identified as one of the molecular markers of Ferroptosis. As displayed in Fig. 3A and B, we found that IL-1β treatment dramatically elevated mRNA and protein levels of Acsl4 in the HCCs, while Fer-1 treatment dramatically depleted the mRNA and protein levels of Acsl4 in both the normal HCCs and IL-1β-treated HCCs. Additionally, the immunofluorescence showed the same results as PCR and Western blot (Fig. 3C).

### Acsl4 knockdown neutralized the ferroptosis progression in the IL-1β-treated HCCs

**Fig. 4:**
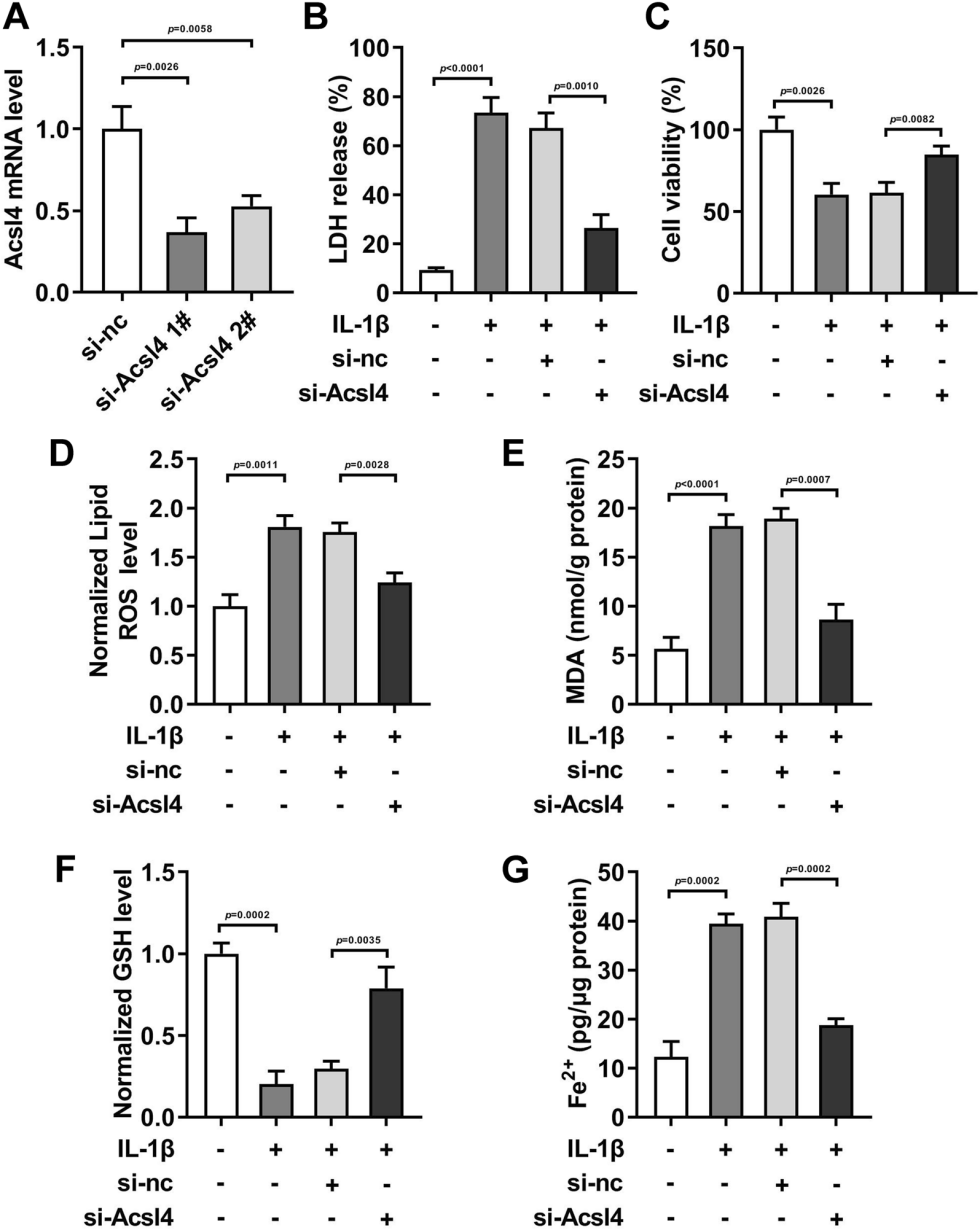
Acsl4 knockdown neutralized the ferroptosis progression in the IL-1β-treated HCCs. Transfection efficiency of si-Acsl4 was analyzed by RT-qPCR (**A**). After IL-1β and si-Acsl4 treatment in the HCCs, the LDH release **B** was measured with LDH Toxicology Kit. Cell viability **C** was detected by CCK-8 assay. The ROS (**D**), MDA (**E**), GSH (F), Fe^2+^ **G** levels were assessed with Corresponding kits

As ACSL4 was significantly upregulated in IL-1beta-induced ferroptosis, we next checked whether ACSL4 silencing could inhibit the ferroptosis. After si-Acsl4 transfection, the Acsl4 levels were prominently depleted (Fig. 4A). The transfection efficiency of si-Acsl4 1# (*P* = 0.0026) was higher than that of si-Acsl4 2# (*P* = 0.0058). Therefore, si-Acsl4 1# was used for the next experiments. Then we found that in the IL-1β-treated HCCs, after si-Acsl4 transfection, the LDH release (Fig. 4C, *P* = 0.0010), ROS (Fig. 4E, *P* = 0.0028), MDA (Fig. 4D, *P* = 0.0057), and Fe^2+^ levels (Fig. 4H, *P* = 0.0005) were dramatically depleted, while cell viability (Fig. 4D, *P* = 0.0082) and GSH levels (Fig. 4G, *P* = 0.0035) were dramatically enhanced.

### Acsl4 knockdown neutralized the roles of IL-1β in the levels of ferroptosis-related genes

**Fig. 5:**
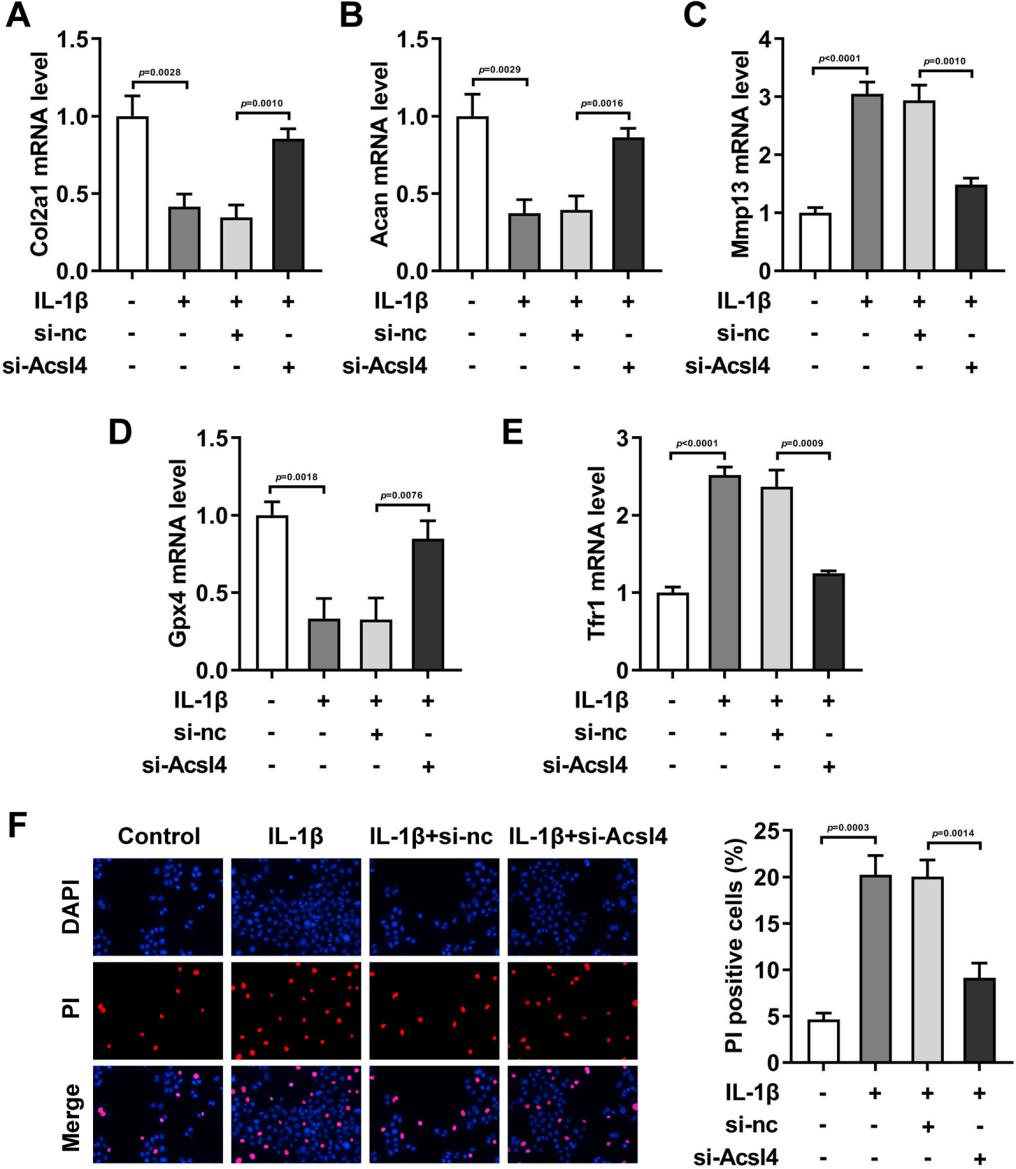
Acsl4 knockdown neutralized the roles of IL-1β in the levels of ferroptosis-related genes. After IL-1β and si-Acsl4 treatment in the HCCs, the mRNA levels of Col2a1 (**A**), Acan (**B**), Mmp13 (**C**), Gpx4 (**D**) and Tfr1 **E** were determined by RT-qPCR assay. (F) The cell death was analyzed by PI staining

Subsequently, we found that in the IL-1β-treated HCCs, after si-Acsl4 transfection, the mRNA levels of Col2a1 (Fig. 5A, *P* = 0.0010), Acan (Fig. 5B, *P* = 0.0016), and Gpx4 (Fig. 5D, *P* = 0.0076) were prominently elevated, while mRNA levels of Mmp13 (Fig. 5C, *P* = 0.0010), Tfr1 (Fig. 5E, *P* = 0.0009), and PI-positive cells (Fig. 5F, *P* = 0.0014) were prominently depleted.

### Sp1 regulated the Ascl4 levels in the IL-1β-treated HCCs

**Fig. 6:**
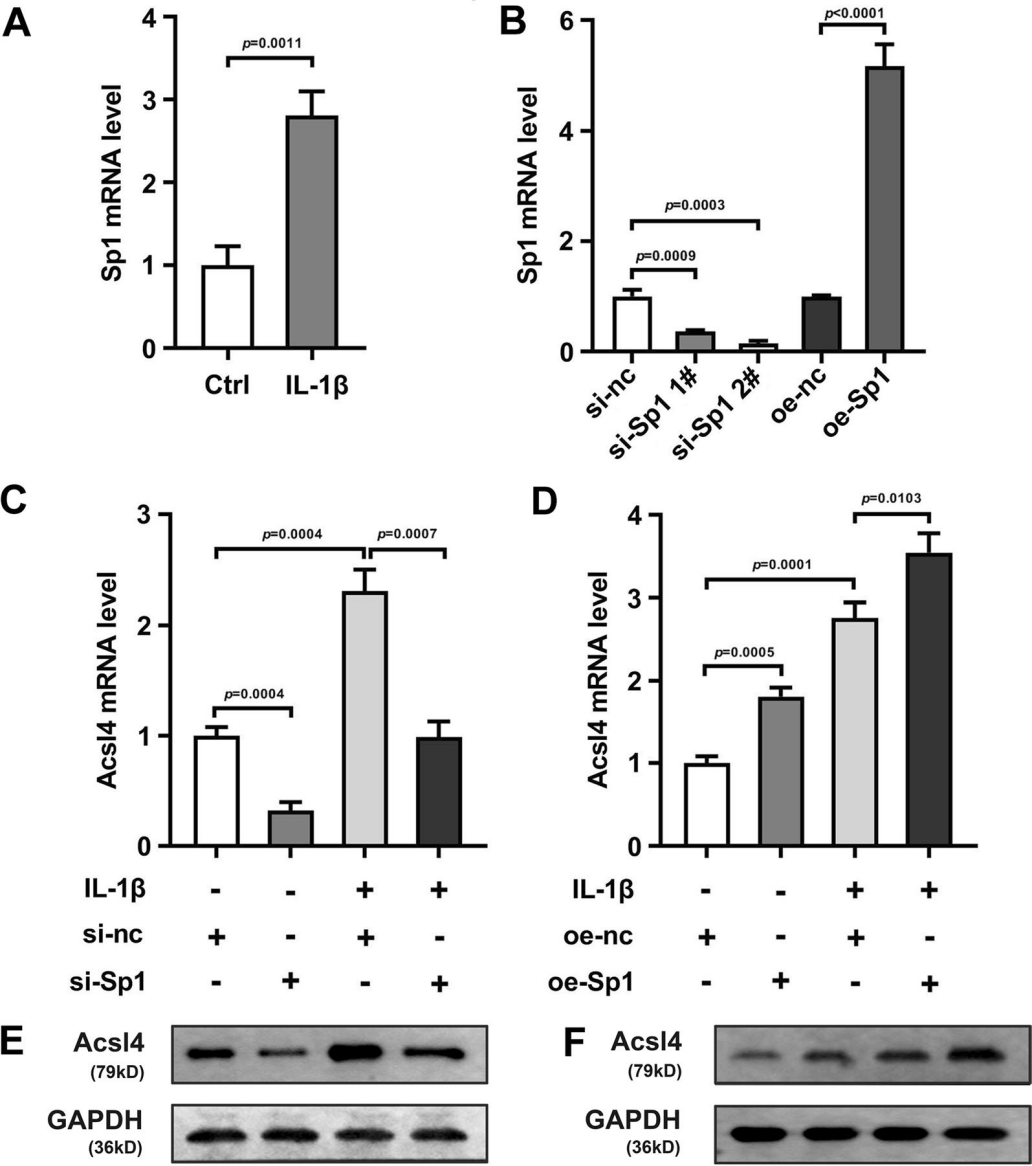
Sp1-regulated Ascl4 levels in the IL-1β-treated HCCs. Sp1 levels in the IL-1β-treated HCCs were detected by RT-qPCR assay (**A**). Transfection efficiency of si-Acsl4 and oe-Sp1 was analyzed by RT-qPCR (**B**). After si-Sp1 **C**, **E** or oe-Sp1 **D** and **F** transfection, the Ascl4 levels in the IL-1β-treated HCCs were detected by RT-qPCR and Western blot

**Fig. 7:**
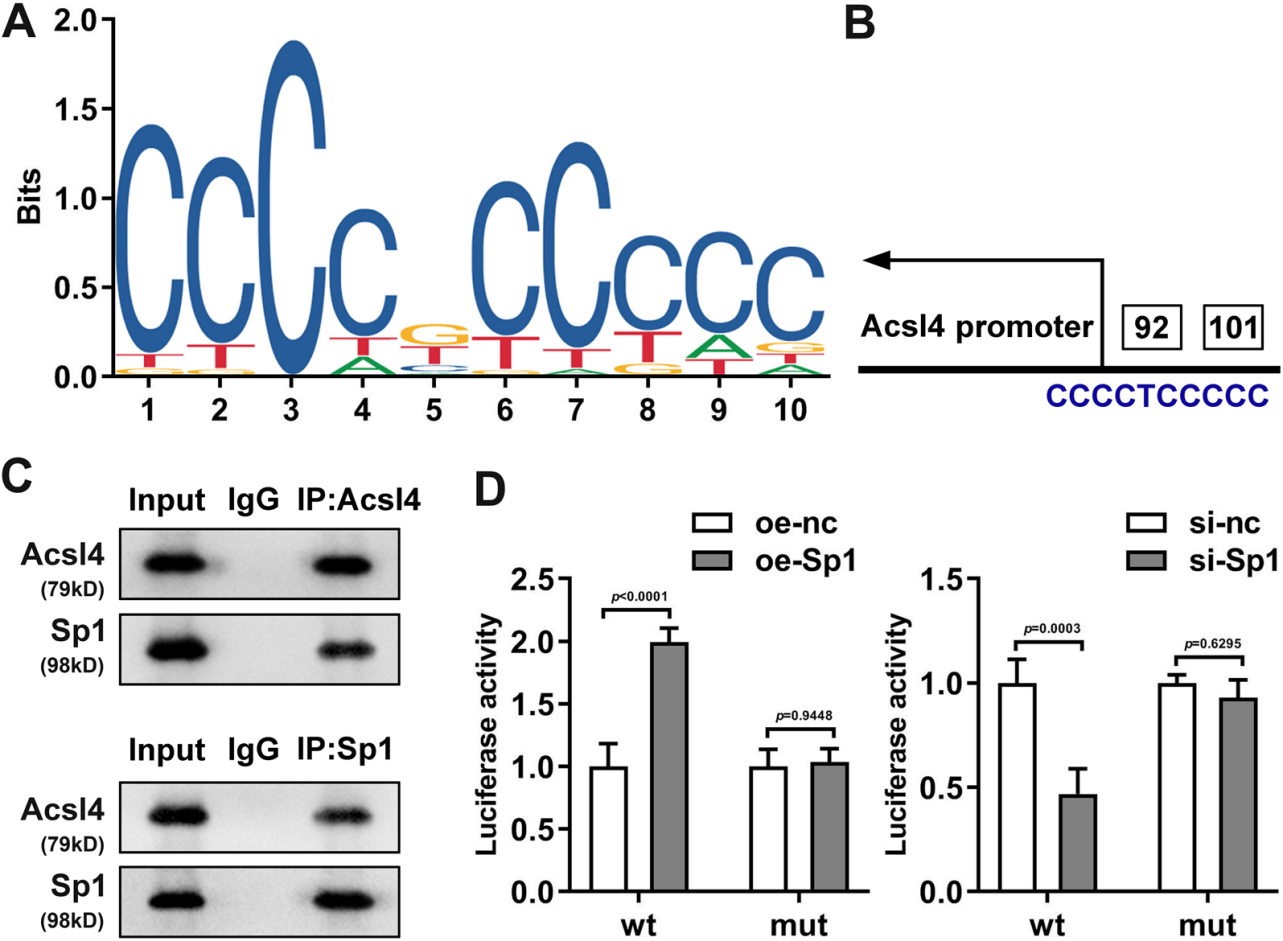
Acsl4 is a direct target gene of Sp1. **A**, **B** Predicted promoter region of Acsl4. The combination between Ascl4 and Sp1 was demonstrated by CO-IP (**C**) and double luciferase report **D** assays

Then we found that IL-1β-treated significantly increased the Sp1 levels in the HCCs (Figure 6A, *P* = 0.00110). After si-Sp1 transfection, the Sp1 levels were declined. The transfection efficiency of si-Sp1 1# (*P* = 0.0003) was higher than that of si-Sp1 2# (*P* = 0.0009). Therefore, si-Sp1 1# was used for the next experiments. Oe-Sp1 transfection prominently elevated the Sp1 levels (Fig. 6B, *P* < 0.0001). Then we found that si-Sp1 transfection dramatically attenuated Acsl4 levels in both normal HCCs (Fig. 6C, *P* = 0.0004) and IL-1β-treated HCCs (Fig. 6C, *P* = 0.0007) at mRNA and protein levels (Fig. 6E). Meanwhile, oe-Sp1 transfection dramatically elevated the Acsl4 levels in both normal HCCs (Fig. 7D, *P* = 0.0005) and IL-1β-treated HCCs (Fig. 7D, *P* = 0.0103) at mRNA and protein levels (Fig. 6F).

### ACSL4 was targeted by Sp1

After confirming the function of ACSL4 in IL-1beta-induced ferroptosis in HCCs, we started to think that how ACSL4 was upregulated. As is well known, gene expression could be enhanced by transcriptional or post-transcriptional regulation. Here, we focused on the transcriptional regulation of ACSL4 in HCCs. First, bioinformatics analysis (https://jaspar.genereg.net/) was performed to predict the transcription factors targeting ACSL4. As showed by the results, the sequence between − 91 and − 101 bp in the Acsl4 promoter region was predicted to bind with Sp1 (Fig. 7A, B). Additionally, the CO-IP assay confirmed the combination between ACSL4 and Sp1 (Fig. 7C). To further confirm the interaction between Sp1 and ACSL4, we carried out luciferase assay. It was found that oe-Sp1 dramatically enhanced the luciferase activity of the WT-Acsl4, but did not change that of mut-ACSL4.(Fig. 7D, *P* < 0.0001), while si-Sp1 dramatically declined it (*P* = 0.0003).

## Discussion

Here, we demonstrated that IL-1β treatment induced the occurrence of ferroptosis in HCCs. ACSL4 knockdown alleviated the injury of HCCs induced by IL-1β. The upregulation of ACSL4 was mediated by transcriptionally activation of Sp1.

The main function of chondrocytes is to maintain the integrity of body cartilage and enable articular cartilage to obtain sufficient weight-bearing energy [21]. Articular chondrocyte apoptosis plays an important role in the progression of OA. Relevant research showed that there was excessive apoptosis of chondrocytes in the articular cartilage of OA [22, 23]. Ferroptosis is a newly defined programmed cell death process different from apoptosis and autophagy, which is characterized by the abnormal increase of intracellular lipid oxygen-free radicals [24]. Previous studies have demonstrated that ferroptosis played an critical role in OA progression. For example, Yao et al. [25] found that Fer-1 treatment might be a promising therapeutic tools for the OA through inhibiting the ferroptosis development. Zhou et al. [26] proved that D-mannose played a chondroprotective role and relieved the OA progression through mitigating the sensitivity of chondrocytes to ferroptosis. Similarly, our research also confirmed that ferroptosis occurred in the IL-1β-treated HCCs, which was manifested as the increase of ROS, MDA, and Fe^2+^ levels and the decrease of GSH levels. Fer-1 treatment neutralized it. Thus, the importance of ferroptosis in the development of OA is self-evident. However, the mechanism of ferroptosis-related genes wasn’t clarified.

As the first step in fatty acid metabolism, Acsl4 catalyzes the synthesis of fatty acyl CoA in vivo and activates long-chain polyunsaturated fatty acids to participate in the synthesis of membrane phospholipids. However, long-chain unsaturated fatty acids on these membranes are often oxidized, resulting in ferroptosis [27]. TfrI is a membrane protein transferrin receptor on the cell membrane. When ferroptosis occurs, it transfers Fe^3+^ into cells and localizes to endosomes when ferroptosis occurs [28, 29]. As a sensor of oxidative stress and cell death signals, the decrease of Gpx4 expression will lead to the significant increase of ROS in vivo, which is considered to be an important target to trigger ferroptosis program [30]. Additionally, Col2a1 mutation can lead to orthopedic diseases such as insufficient cartilage formation, osteoarthritis, congenital spondyloepiphyseal dysplasia, etc. Acan and Col2a1 are the main components of cartilage matrix [31]. IL-1β can stimulate the synthesis of matrix metalloproteinases (MMPs), which can promote the degradation of cartilage matrix [32]. This study found that IL-β stimulation prominently induced the decrease of Col2a1, Acan, and Gpx4, and the increase of Mmp13 and Tfr1 in the HCCs. And Fer-1 treatment neutralized it. Interestingly, we found that Fer-1 treatment decreased the Acsl4 levels in both the normal HCCs and IL-1β-treated HCCs, while other related genes levels in the normal HCCs showed no difference after Fer-1 treatment. Therefore, we speculated that Acsl4 might be the key in the IL-1β-treated HCCs. After the performance of rescue experiment, we found Acsl4 knockdown prominently neutralized the injury of HCCs induced by IL-1β stimulation. All these findings indicated Acsl4-mediated ferroptosis promoted the OA progression.

Subsequently, after bioinformatic analysis, we found the sequence between − 91 and − 101 bp in the Acsl4 promoter region is predicted to bind Sp1. As a classical transcription factor, Sp1 has been demonstrated to bind to the target gene promoter through the DNA binding domain to activate or inhibit the transcription of a variety of target genes, such as CyclinD, E-cadherin, transforming growth factor β, histone deacetylation, etc., thereby regulating cell cycle, angiogenesis, apoptosis, tumorigenesis and development, chromatin remodeling and other biological processes [16, 33]. However, the role of Sp1 in OA development remains unclear. This study further confirmed that Sp1 was located in the nucleus of HCCs. Sp1 knockdown declined the Acsl4 levels and Sp1 overexpression elevated it. Double luciferase report further confirmed the relationship between Sp1 and Acsl4. Li et al. [9] found Sp1 enhanced the Acsl4 expressions through binding to the promoter region in intestinal ischemia/reperfusion injury mice, which was similar to our results. However, there are still some limitations in this study.

In conclusion, this study demonstrated that Acsl4-mediated ferroptosis played a critical role in OA progression. Sp1 can elevated the Acsl4 levels through binding to the ACSL4 promoter region. These results guided us to extend ferroptosis inhibition to OA treatment. Our study is a basic research and does not involve in vivo experiments. In the future, we will carry out in vivo experiments for further research.

## Data Availability

The datasets used and/or analyzed during the current study are available from the corresponding author on reasonable request.
